# Supplementary figures and images for: E3 ubiquitin ligase Deltex facilitates the expansion of Wingless gradient and antagonizes Wingless signaling through a conserved mechanism of transcriptional effector Armadillo/β-catenin degradation
Source: eLife. 2024 Jun 20;12:RP88466. doi: 10.7554/eLife.88466 (PMC11189633; doi:10.7554/eLife.88466)

## Slide 1
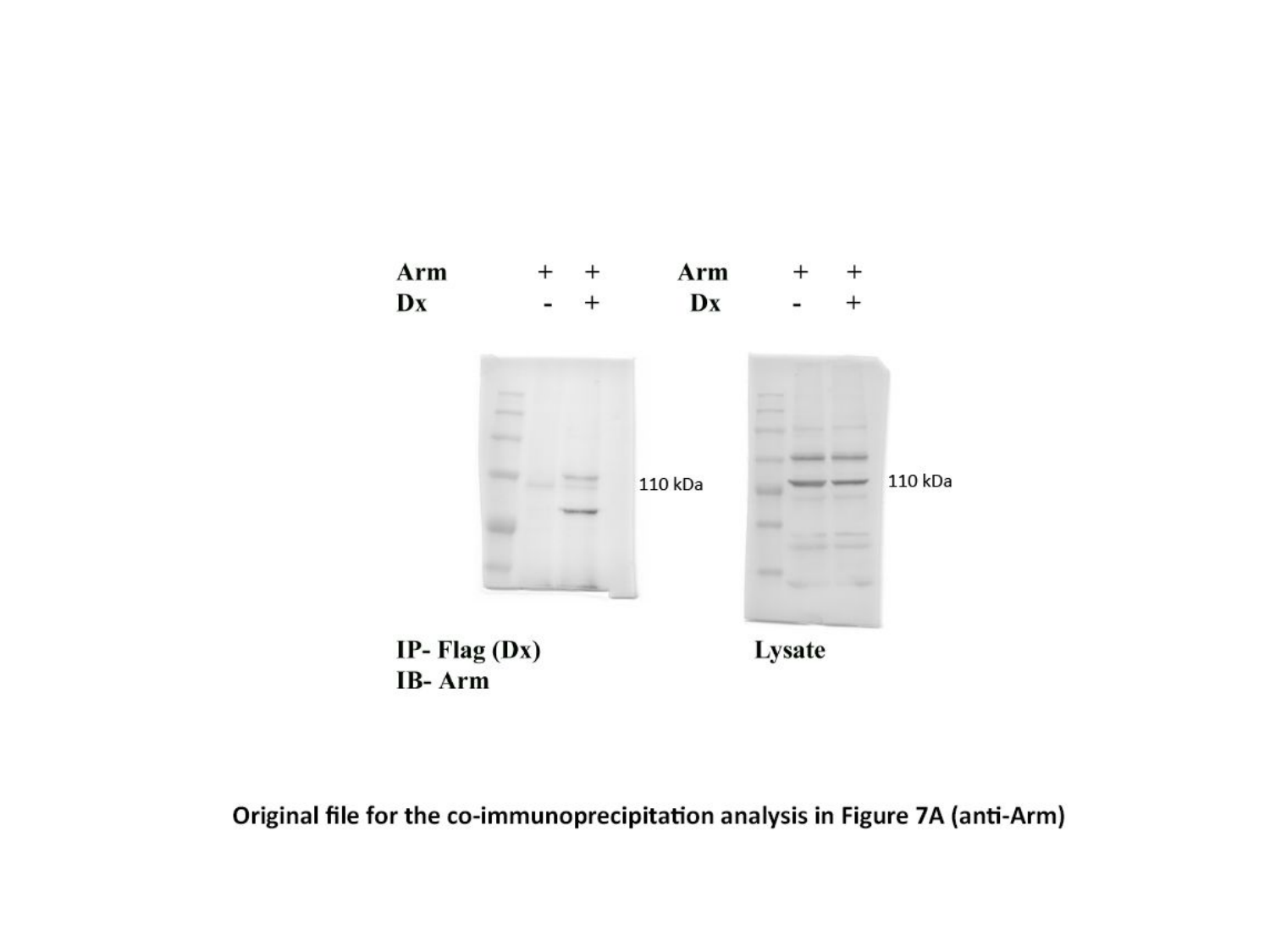

Supplement: Figure 7—source data 1. [file elife-88466-fig7-data1.pptx]

## Slide 1
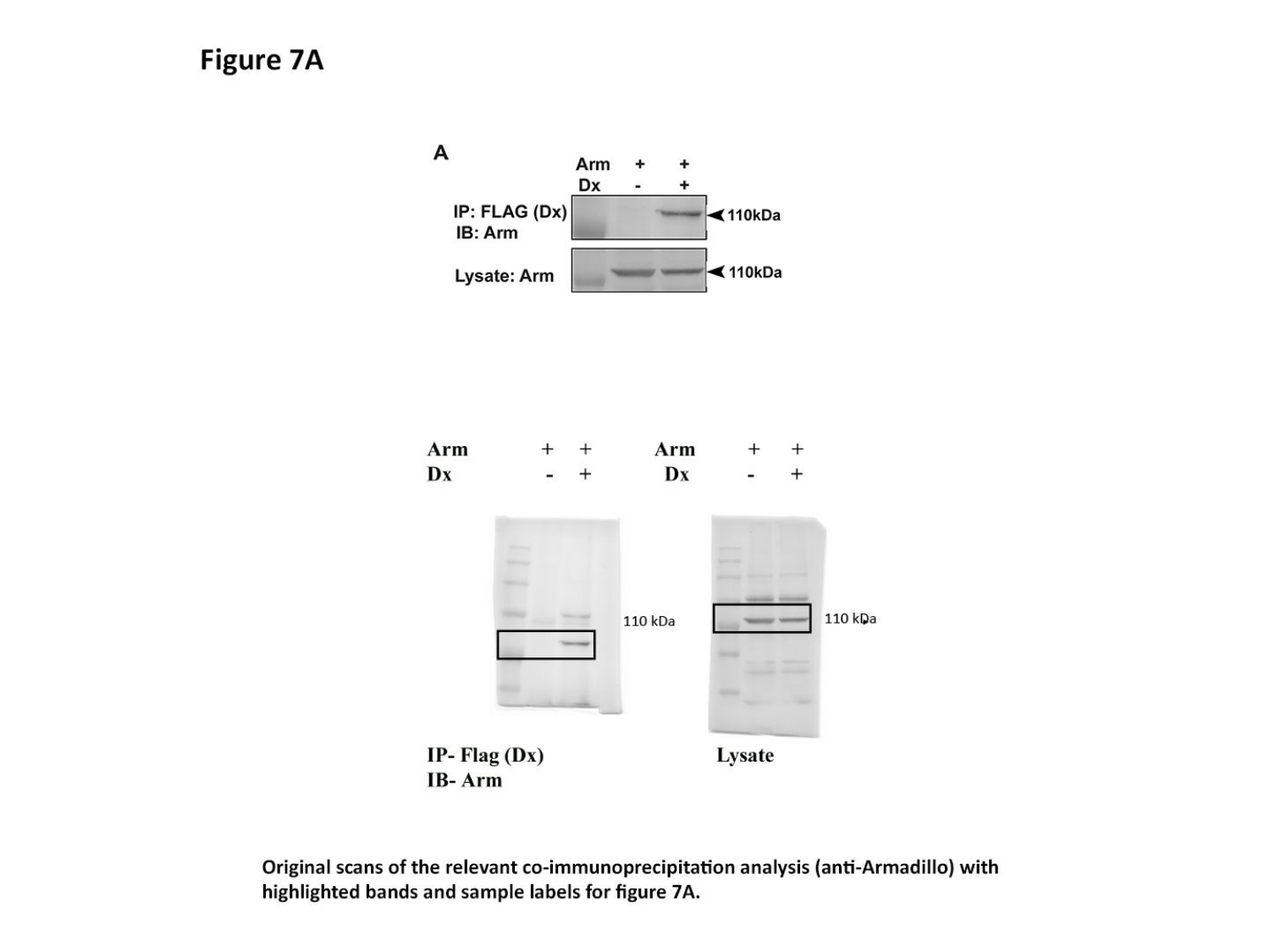

Supplement: Figure 7—source data 2. [file elife-88466-fig7-data2.pptx]

## Slide 1
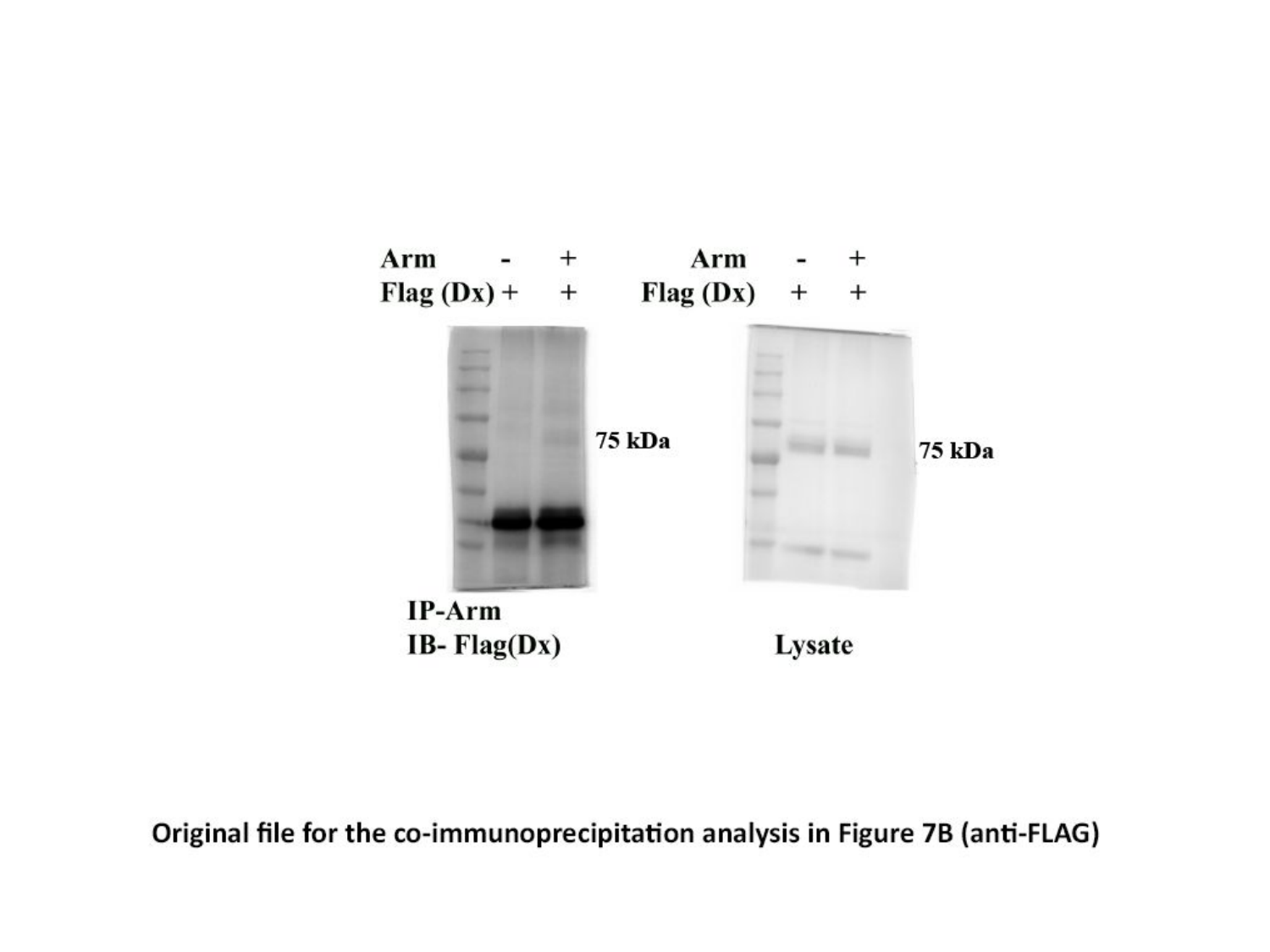

Supplement: Figure 7—source data 3. [file elife-88466-fig7-data3.pptx]

## Slide 1
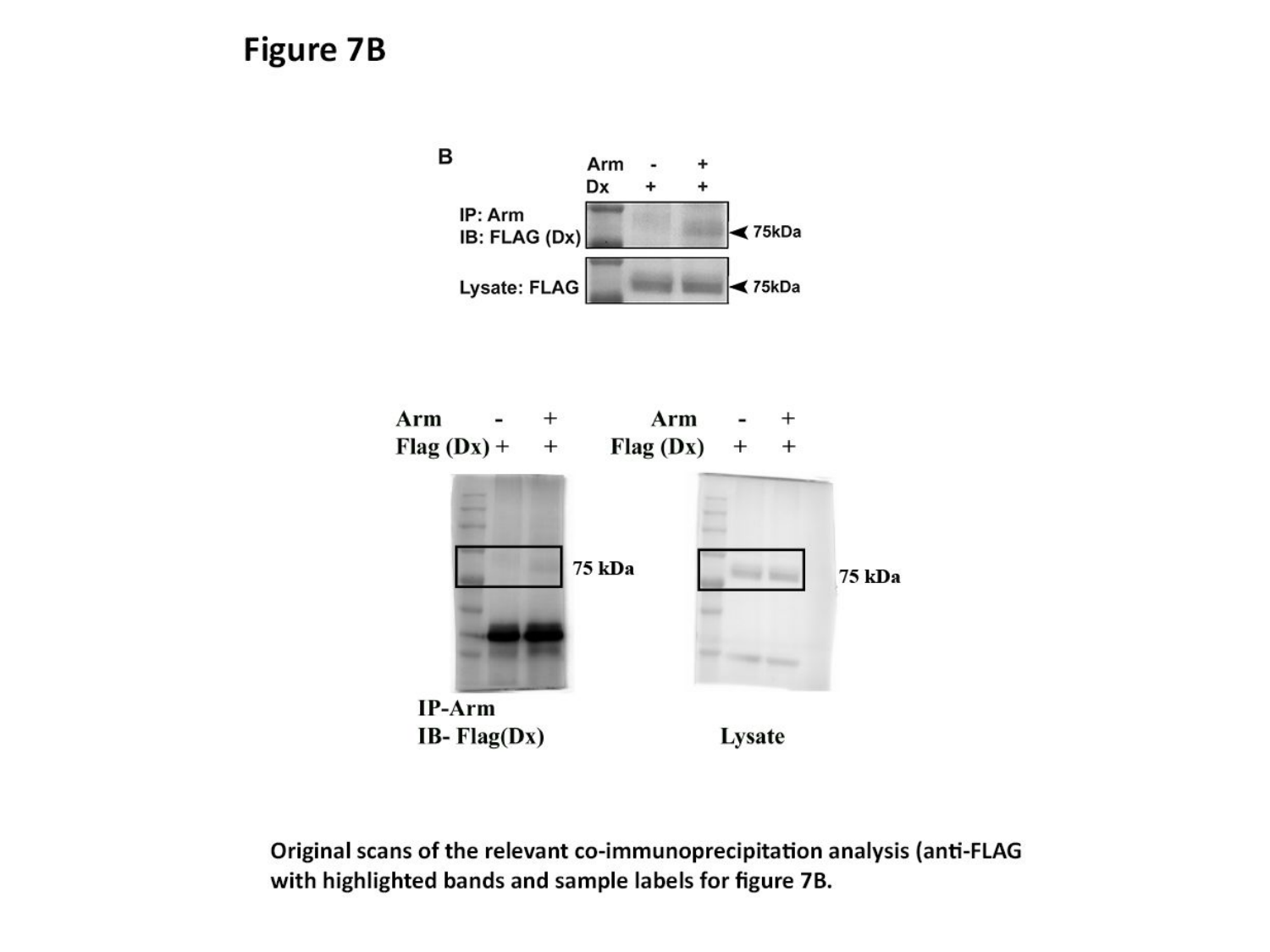

Supplement: Figure 7—source data 4. [file elife-88466-fig7-data4.pptx]

## Slide 1
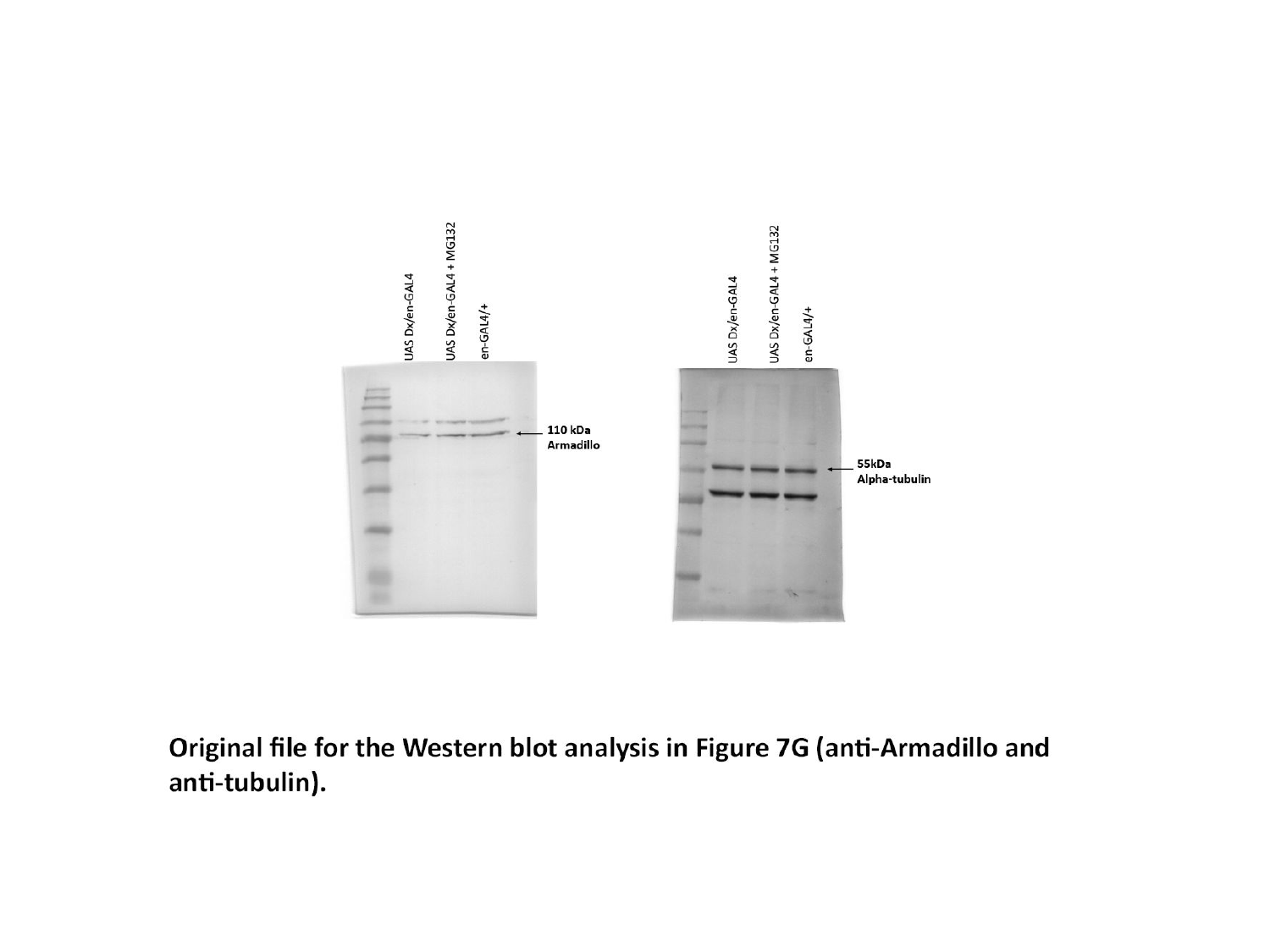

Supplement: Figure 7—source data 5. [file elife-88466-fig7-data5.pptx]

## Slide 1
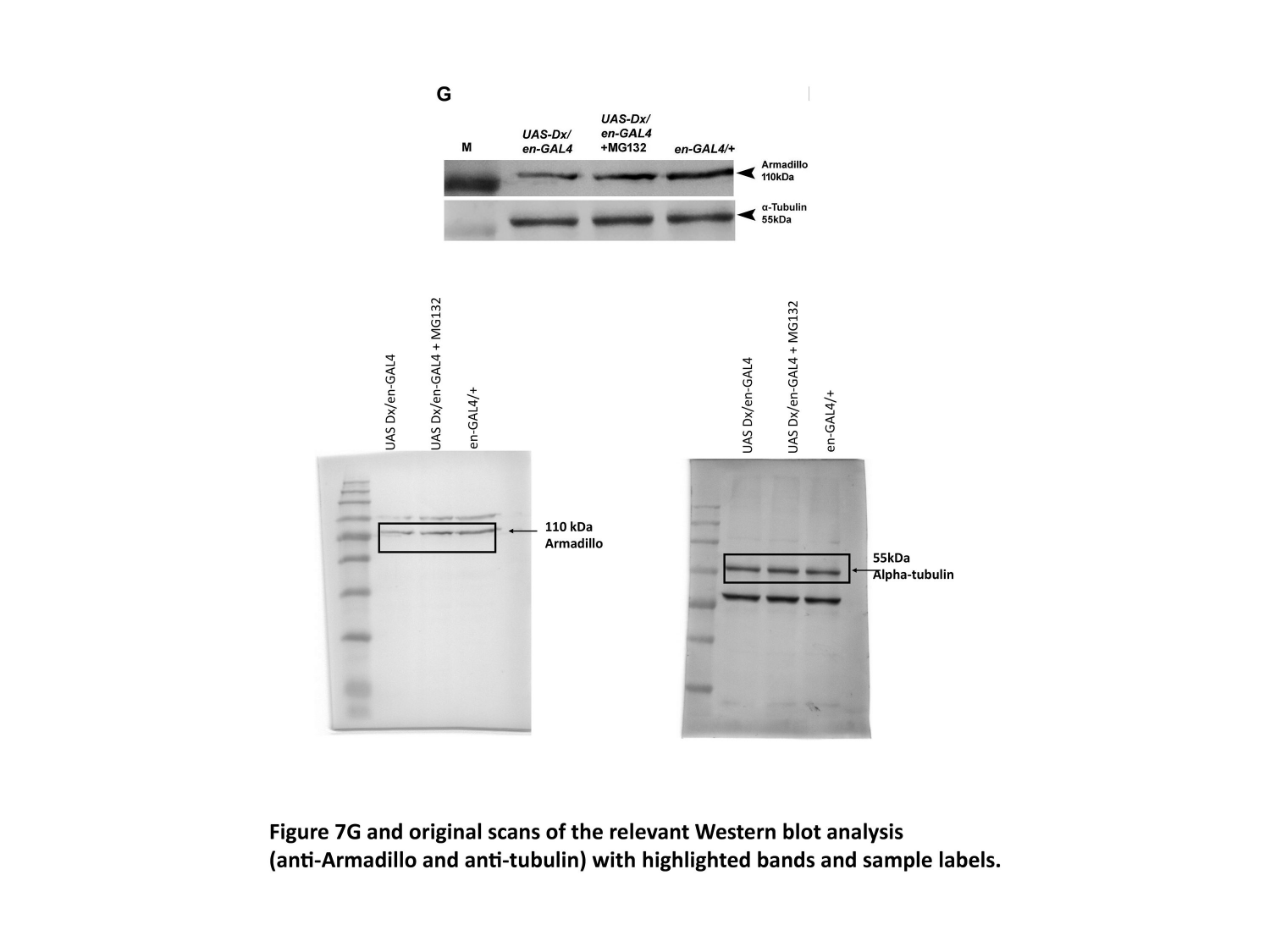

Supplement: Figure 7—source data 6. [file elife-88466-fig7-data6.pptx]

## Slide 1
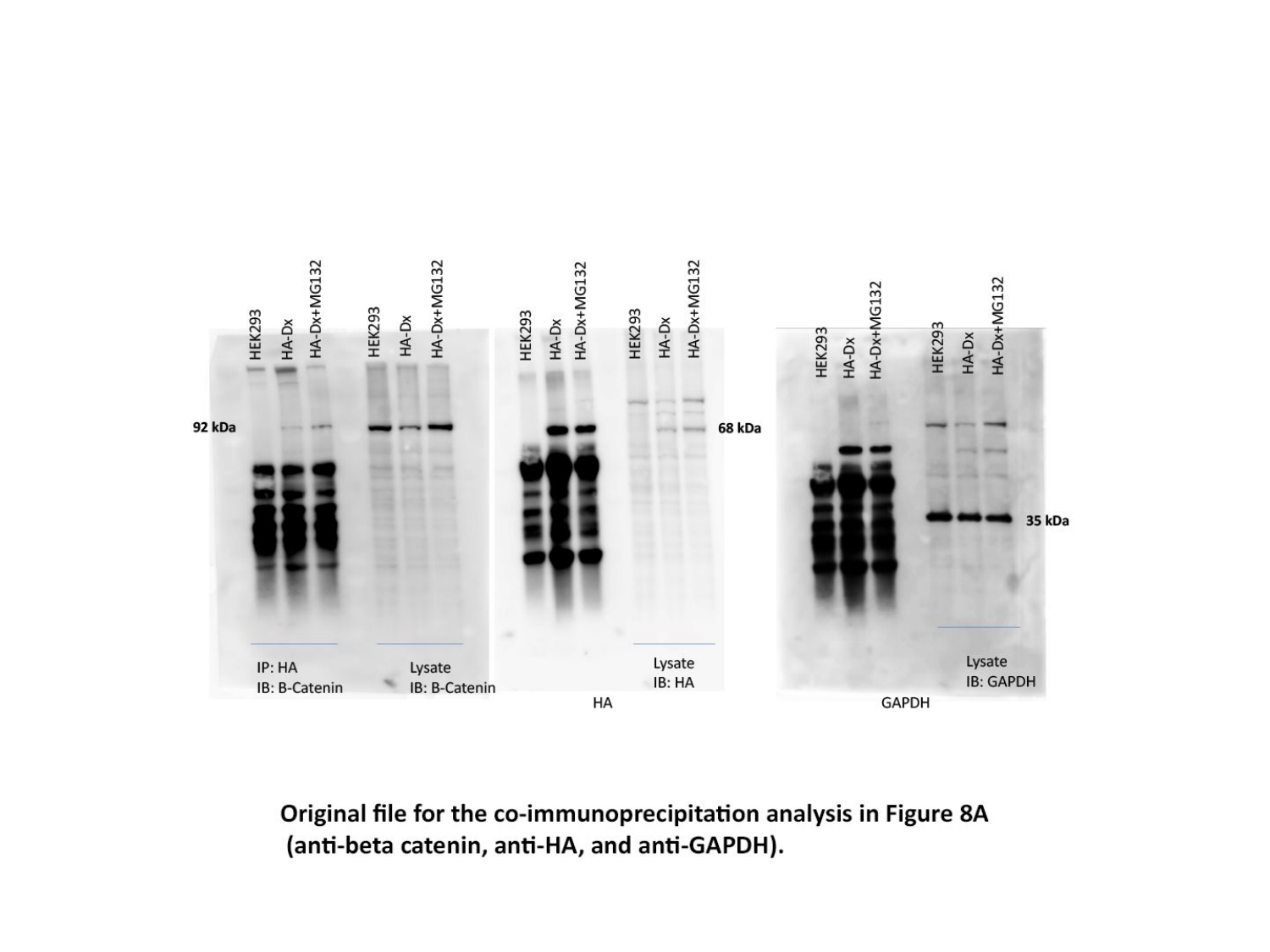

Supplement: Figure 8—source data 1. [file elife-88466-fig8-data1.pptx]

## Slide 1
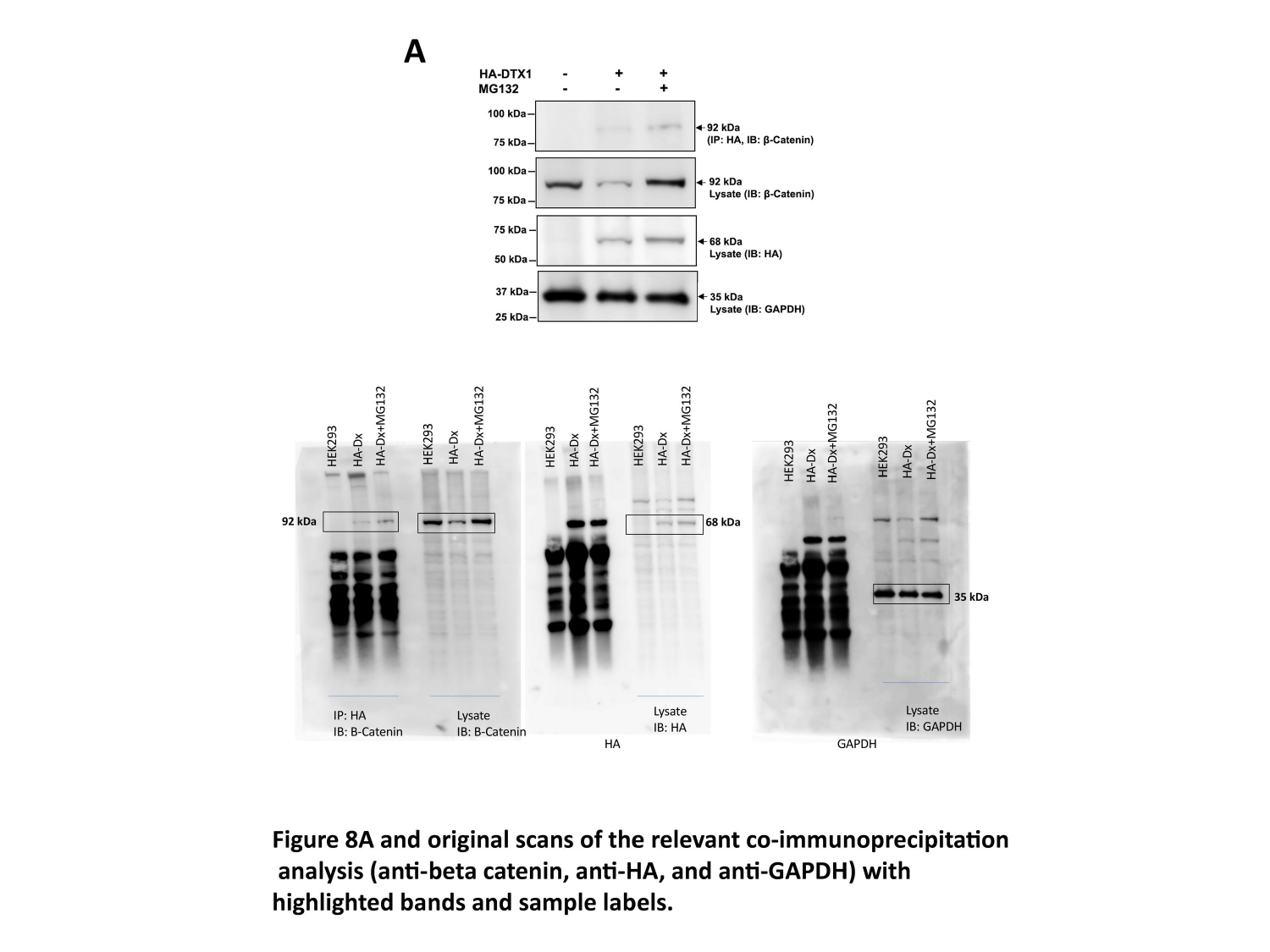

Supplement: Figure 8—source data 2. [file elife-88466-fig8-data2.pptx]

## Slide 1
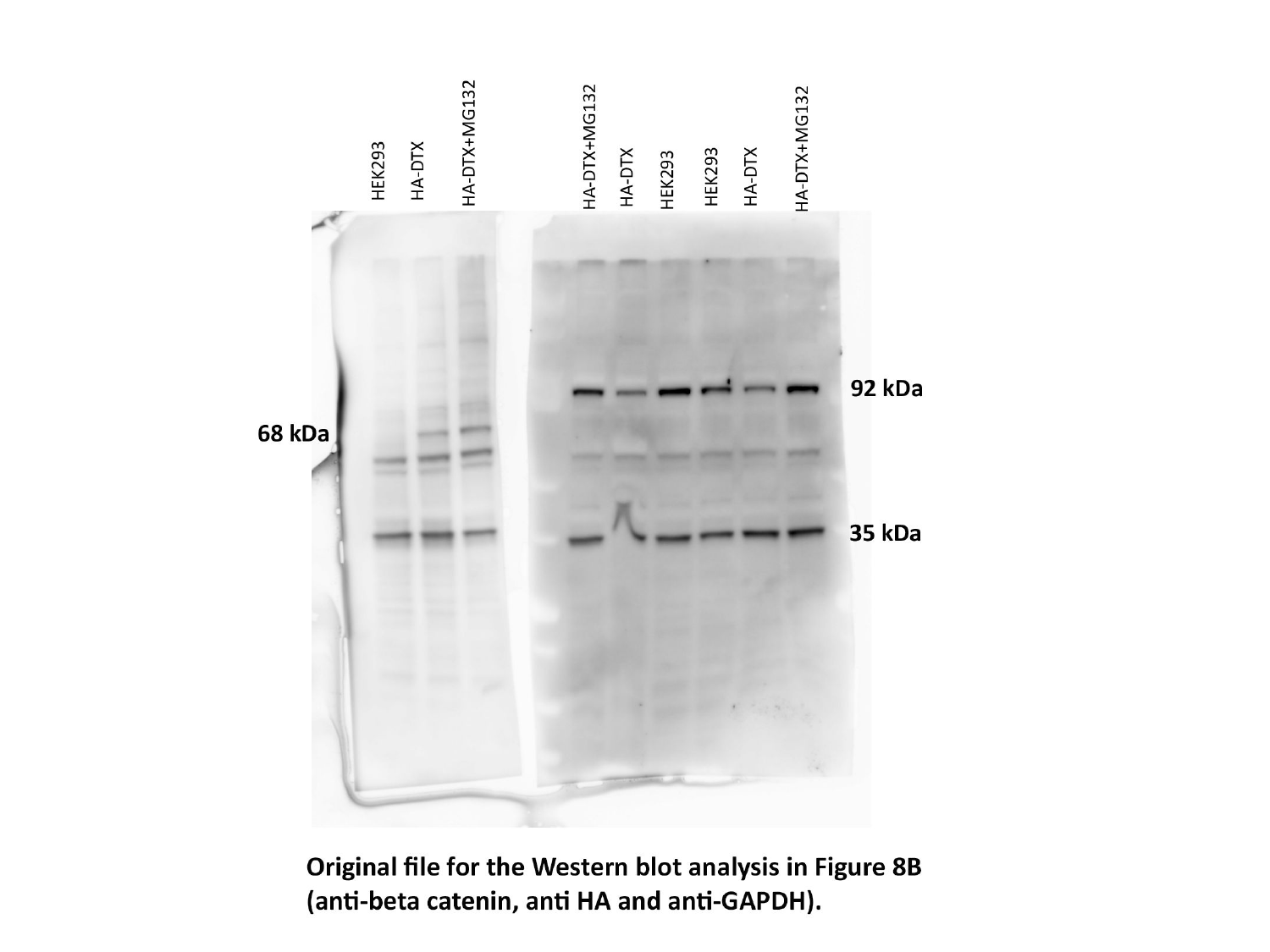

Supplement: Figure 8—source data 3. [file elife-88466-fig8-data3.pptx]

## Slide 1
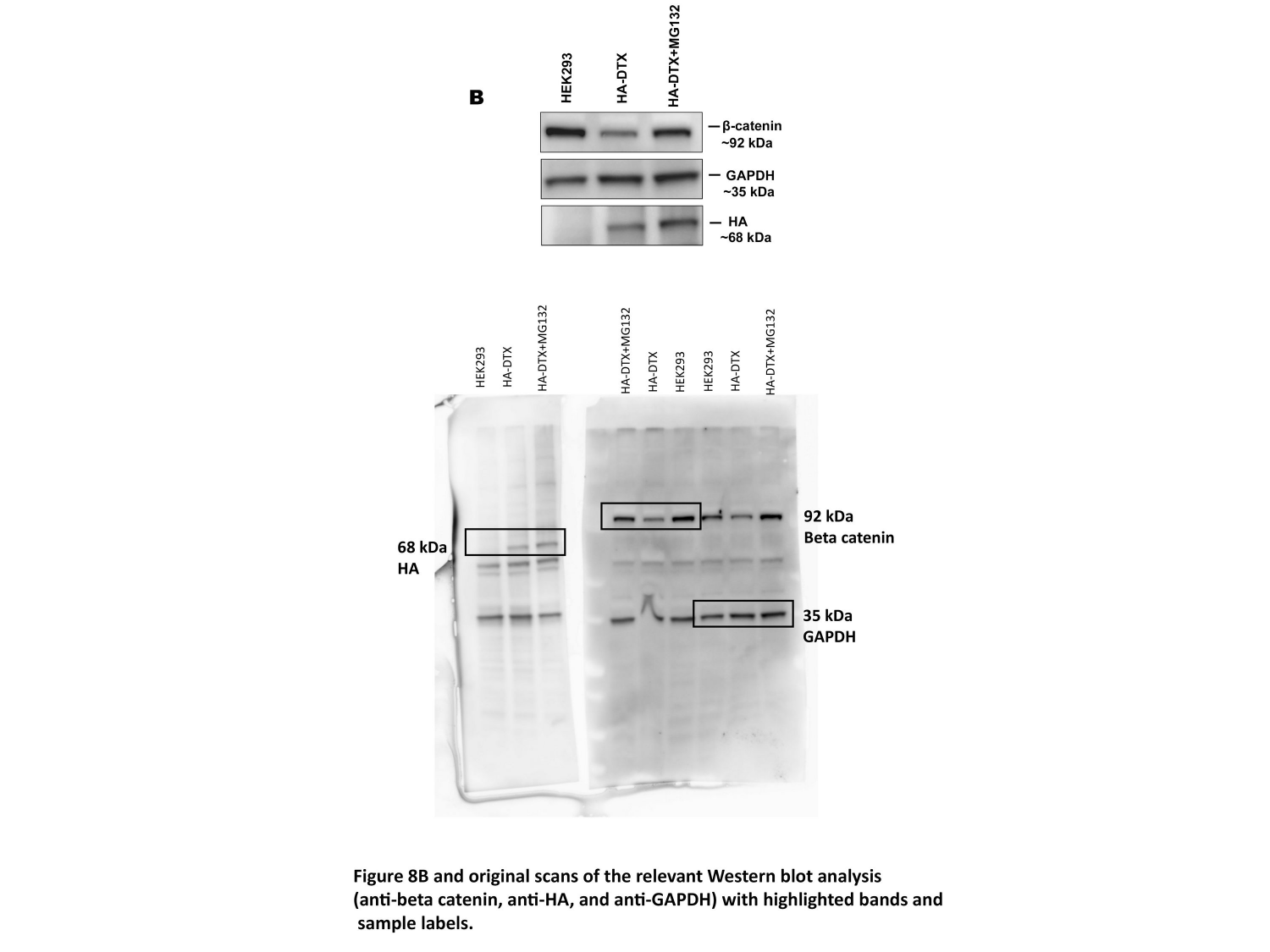

Supplement: Figure 8—source data 4. [file elife-88466-fig8-data4.pptx]
